# Museum visitors’ acceptance of digital services in China and Russia: Extending UTAUT2 with individual-level cultural values

**DOI:** 10.1371/journal.pone.0357563

**Published:** 2026-11-02

**Authors:** Jing Chen, Xiaobin Li, Chuanhua Zhu, Marina Biryukova

**Affiliations:** 1 College of Landscape Architecture and Arts, Northwest A&F University, Yangling, Shaanxi, China; 2 Institute of Philosophy, St Petersburg University, St Petersburg, Russia; 3 School of Design, Jiangnan University, Wuxi, China; University of Trento: Universita degli Studi di Trento, ITALY

## Abstract

Museum digital services have become an important means of supporting visitors’ access to information, understanding of exhibition content, and broader museum experiences. However, the mechanisms underlying visitors’ acceptance of these services may vary across visitor groups. Drawing on a context-adapted Unified Theory of Acceptance and Use of Technology model (UTAUT2), this study incorporates three individual-level cultural values—collectivism orientation, uncertainty avoidance, and long-term orientation—to examine acceptance intention toward museum digital services in China and Russia. The model was tested using partial least squares structural equation modeling (PLS-SEM) with survey data from China (n = 316) and Russia (n = 307). Measurement invariance of composites (MICOM) and PLS multigroup analysis (PLS-MGA) were conducted to assess between-sample differences. Performance expectancy, effort expectancy, social influence, facilitating conditions, and hedonic motivation all had significant positive effects on acceptance intention in both samples. The PLS-MGA results showed no significant between-sample differences in the effects of performance expectancy or social influence. Hedonic motivation had a significantly stronger effect in the Chinese sample, whereas effort expectancy and facilitating conditions had stronger effects in the Russian sample. The latter two differences should nevertheless be interpreted cautiously because compositional invariance was not established for these constructs. Collectivism strengthened the effect of social influence, uncertainty avoidance strengthened the effects of effort expectancy and facilitating conditions, and long-term orientation strengthened the effects of performance expectancy and hedonic motivation. None of the five moderating effects differed significantly between the two samples. These findings show that acceptance intention toward museum digital services is jointly shaped by functional benefits, ease of use, social cues, available support, experiential value, and visitors’ individual cultural value orientations. The study provides evidence of both shared acceptance mechanisms and sample-specific path differences and offers a basis for visitor segmentation and the refinement of museum digital services.

## Introduction

In recent years, digital media, immersive technologies, and interactive design have become an increasing part of museum exhibition and public service systems, with online exhibitions, websites, mobile guides [1], and AR/VR-based experience formats being increasingly integrated into the ways visitors engage with cultural heritage [2,3]. Rather than functioning as isolated technological applications, these digital tools are increasingly being incorporated into broader museum service systems that support communication, learning, interaction, and cultural participation before, during, and after the visit. This shift means that museum digitalization should be understood not only as the adoption of new technologies, but also as the reorganization of visitor-oriented services and experiences. However, the wider availability of digital services does not necessarily lead to visitor acceptance. When facing similar digital functions, different visitors may reach very different judgments [4]. Therefore, as museum digitalization continues to advance, two central questions are whether visitors are willing to accept these services and whether the formation of such acceptance intention follows different patterns across cultural contexts. In this study, acceptance intention is operationalized as behavioral intention (BI), referring to visitors’ intention to use museum digital services in the future [5]. In technology acceptance research, BI is commonly used to capture the proximal stage through which individual evaluations are translated into actual use behavior [6,7]. Examining acceptance intention is therefore an important step in understanding visitors’ adoption of museum digital services.

Technology acceptance research provides a mature and reusable framework for explaining the adoption of digital services [8]. Among these frameworks, the Unified Theory of Acceptance and Use of Technology (UTAUT) and its extended version, UTAUT2, have been widely used to examine how users form behavioral intentions toward different information systems and digital services [9]. Venkatesh et al. stressed that whether users accept a technology usually depends on their subjective evaluation of expected benefits, required effort, and social context, rather than on technical features alone [10]. In the context of museum digital services, visitors often make such judgments under conditions of limited attention and limited dwell time [11], and their perception of information value, learning effort, and experience will jointly shape whether they are willing to further use such services [12,13]. However, the application of UTAUT2 to museum digital services has mostly been developed within single-country or single-service settings, and less is known about whether the relative importance of these acceptance factors changes across cultural contexts.

Technology acceptance also takes place within a social and cultural environment. Previous research has shown that technology adoption involves not only evaluations of functional benefits and costs but also the influence of cultural context [14]. Because adoption is shaped by both technical attributes and social context, cultural value orientations may further change the relative weight of different antecedents of behavioral intention within the UTAUT2 framework [15]. The same performance benefits, required effort, or social influence may be given different levels of importance under different cultural orientations, which can lead to different responses to the same technology across user groups [16]. Accordingly, this study incorporates Hofstede-derived cultural values into the UTAUT2 model and measures them at the individual level [17] to examine how visitors’ value orientations shape the formation of acceptance intention.

Building on this, the present study draws on samples from China and Russia to examine similarities and differences in museum visitors’ acceptance mechanisms across distinct social and digital service contexts. Both countries are advancing the digitalization of public cultural services, although their implementation pathways differ. Comparing these two digital service environments makes it possible to assess whether the core UTAUT2 factors operate consistently across contexts and to further examine how individual-level cultural values shape the decision cues on which visitors rely when forming acceptance intention.

A review of research on museum digital services reveals two common lines of work. The first starts from technology and experience evaluation and focuses on how immersive technologies such as AR and VR, together with their interactive forms, shape learning, immersion, and user experience, while also identifying possible barriers and limits to use [2,3,18–21]. The second treats digital services as information systems and examines their usability and adoption intention, including evaluations of websites and digital interfaces [1,22,23], as well as studies that use TAM or UTAUT in a single country or a single project to explain the adoption and continued use of services such as mobile applications and virtual exhibition halls [13,24–26]. In addition, UTAUT2 has accumulated a relatively strong evidence base in broader studies of digital consumption and public services, which provides methodological support for its application in specific contexts [9,27–29]. Hofstede’s cultural dimensions have also been combined with UTAUT2 to examine the potential role of culture in technology adoption [16,30,31].

Although research on museum digital services has progressed, gaps remain in understanding visitors’ acceptance mechanisms. First, existing studies often focus on a single technology or service, with limited attention to visitors’ acceptance of the overall digital support embedded in the museum visit. Second, most studies rely on single-country samples, while cross-cultural comparisons often remain descriptive and provide limited explanation of how individual-level cultural values shape the effects of UTAUT2 factors on acceptance intention.

To address these gaps, this study examines the following research questions:

RQ1: How do the key factors in UTAUT2 influence visitors’ acceptance intention toward museum digital services?
RQ2: Do the effects of these factors differ significantly between the Chinese and Russian samples?
RQ3: How do individual-level cultural values moderate the effects of these factors on acceptance intention?

To address these issues, this study focuses on museum visitors’ acceptance intention toward digital services (BI). Within the UTAUT2 framework, collectivism orientation, uncertainty avoidance, and long-term orientation are incorporated as individual-level cultural moderators and tested using Chinese and Russian samples. PLS-SEM is used to examine the direct and moderating effects, while MICOM and PLS-MGA are applied to assess measurement invariance and between-group differences in the structural paths.

This study makes three main contributions. Theoretically, it incorporates individual-level cultural values into a model of museum digital service acceptance and clarifies how these values shape visitors’ reliance on social cues, operating conditions, and experiential value. Methodologically, it combines individual-level cultural measurement with MICOM and PLS-MGA, distinguishing numerical differences in path coefficients from statistically supported between-group differences and avoiding the direct equation of nationality with culture. Practically, the findings provide a basis for visitor segmentation and for improving the functions, operational support, experiential design, and promotion of museum digital services.

## Theoretical background and research model

### Museum digital services

As digital technologies have become increasingly integrated into museum exhibitions and public services, museum digitalization has expanded beyond collection digitization and back-end management to support how visitors access information, interpret exhibitions, participate in activities, and extend their museum experiences [32]. Museum digital services therefore encompass not only individual technologies or devices but also a range of digitally mediated services that support visitor access, interpretation, interaction, and participation. These practices broaden access to museum collections and contribute to knowledge preservation, resource use, and visitor experience [33,34].

In actual visits, these digital functions are often distributed across pre-visit information search, on-site guidance and interaction, and post-visit review and sharing, thereby supporting the full visiting process and its extended experience [35]. Visitors may therefore encounter several forms of digital support during the same museum experience rather than relying on a single isolated technology. As a result, visitors usually form an overall judgment of whether the museum’s digital support is useful, easy to use, and smooth as an experience. Lu et al. also noted that museum technology research has gradually moved beyond early concerns with learning and interaction toward variables that are closer to the adoption process, such as intention, behavior, and experience outcomes [36].

In both the Chinese and Russian contexts, museum digital services have continued to develop under the combined influence of policy support and platform building. In China, plans for cultural relic protection and scientific innovation have clearly called for stronger technology application and public service capacity in the museum sector [37,38]. In Russia, public-facing mobile guides and digital access points are provided through cultural sector projects and public platforms [39]. Given the differences in service provision pathways and platform ecosystems between the two countries, restricting the research object to a single technological entry point would make cross-sample comparison more vulnerable to asymmetries in availability and use scenarios. For this reason, this study uses the term museum digital services to cover mini-programs or mobile applications, online exhibitions, and AR- or VR-based guides, and takes visitors’ perceptions of overall digital support as the unit of analysis.

### Unified theory of acceptance and use of technology

Technology acceptance refers to an individual’s willingness to adopt and use a technology [40]. In this study, behavioral intention (BI) is referred to as acceptance intention and represents visitors’ intention to use museum digital services in the future. Behavioral theory suggests that individual action is often organized first through intention [41], and intention serves as a link among attitude, social norms, and perceived behavioral control [5]. Therefore, although a gap remains between intention and behavior, meta-analytic evidence shows a strong statistical association between the two [42], and in the absence of longitudinal tracking data or usage logs, using BI as the main outcome variable remains methodologically reasonable [43].

The UTAUT family of models offers a relatively unified framework for explaining technology adoption and has been widely used to analyze adoption intention across different technologies and services [44,45]. It has also demonstrated explanatory value for behavioral intention in previous empirical applications [46]. In 2012, Venkatesh et al. extended the research setting from organizational use to consumer and voluntary use by proposing UTAUT2. Beyond the original core paths, the model added hedonic motivation, price value, and habit, which made it more suitable for explaining intention formation in experience-driven and non-mandatory adoption settings [7].

In public-facing digital service settings, BI is often shaped by both instrumental evaluation and experiential evaluation. More importantly, UTAUT offers a useful basis for explaining acceptance in social and cultural contexts, which makes it suitable for studying the digitalization of public cultural services [8,47]. Recent studies have also applied UTAUT-based and related user-acceptance models to digital museums and cultural heritage services, showing that functional value, ease of use, and experiential factors may contribute to shaping users’ acceptance and usage intentions [13,48,49]. However, these studies have generally focused on a particular technology, digital platform, or national context, leaving the cross-cultural acceptance of broader museum digital support less fully examined.

Based on the specific context of museum digital services, this study assessed the contextual suitability of the UTAUT2 variables. Price value and habit were excluded because of their limited applicability to the present study. Price value reflects the trade-off between the perceived benefits of a technology and its monetary cost [7]. However, most of the museum digital services covered by the questionnaire, including mobile guides, online exhibitions, and AR/VR-based experiences, were not presented as separately priced products, leaving respondents without a consistent basis for evaluating this trade-off. Habit is more applicable to technologies used frequently and under relatively stable conditions [50], whereas museum digital services are often encountered during particular visits and may vary across museums and visiting situations, making stable habitual use difficult to assess. Although some services may involve indirect costs or repeated use, these conditions do not apply consistently across the overall service context examined here.

Therefore, on the basis of the context-adapted UTAUT2 model, this study retained performance expectancy (PE), effort expectancy (EE), social influence (SI), facilitating conditions (FC), and hedonic motivation (HM) to explain visitors’ acceptance intention (BI) toward museum digital services. Performance expectancy (PE) reflects visitors’ judgment of whether digital services can generate benefits for the museum visit [12], and existing studies suggest that when users believe a technology can deliver clear benefits, their acceptance intention tends to become stronger [51]. Effort expectancy (EE) refers to perceived ease of use, that is, visitors’ perception of the learning and operational costs involved in using digital technology. Easy-to-use interface design can reduce technical barriers and strengthen visitors’ willingness to use digital services, particularly in heritage visits and on-site guidance settings where immediate interaction is required [52]. Social influence (SI) concerns the extent to which normative cues, such as peer suggestions, social media discussions, or the atmosphere on site, shape intention. The use of museum digital services is not a purely private technical act, but often takes place in visiting situations with clear social visibility. Related studies also show a positive relationship between social influence and behavioral intention [26,48], which makes this factor meaningful in cross-sample comparison. Facilitating conditions (FC) concern whether external resources such as network access, devices, on-site guidance, and staff support are available. They affect not only whether the service can be used, but also how visitors assess usage risk and time costs. In consumer settings, differences in facilitating conditions are more likely to influence intention and use [13]. HM reflects the enjoyment, fun, and sense of immersion experienced during use. Studies of museum VR and related digital experiences have identified enjoyment and immersion as important factors in users’ evaluations, satisfaction, and continued engagement [49]. The retention of HM is particularly relevant because museum digital services combine informational functions with cultural, aesthetic, and experiential engagement.

## Based on these considerations, the following hypotheses are proposed

H1a: Performance expectancy (PE) has a positive effect on acceptance intention (BI).
H1b: Effort expectancy (EE) has a positive effect on acceptance intention (BI).
H1c: Social influence (SI) has a positive effect on acceptance intention (BI).
H1d: Facilitating conditions (FC) have a positive effect on acceptance intention (BI).
H1e: Hedonic motivation (HM) has a positive effect on acceptance intention (BI).

### Hofstede’s cultural dimensions

Individuals’ interpretations of social norms, judgments of uncertainty in technology use, and evaluations of long-term benefits are often influenced by their cultural value orientations. Srite and Karahanna incorporated individual-level cultural values into a technology acceptance framework and showed that individuals’ endorsement of particular cultural values can alter the way factors such as social influence affect behavioral intention [15]. Subsequent research integrating cultural dimensions with UTAUT2 has also suggested that cultural values do not necessarily determine technology acceptance directly; rather, they may change the strength of the relationships between different antecedents and intention formation [30].

Hofstede conceptualized national culture along six dimensions, providing a structured framework for examining the relationship between culture and technology acceptance [53]. However, Hofstede’s national culture scores primarily reflect aggregate societal tendencies and cannot be assumed to represent the actual value orientations of every individual within a country. Directly inferring individual-level cultural characteristics from national-level cultural patterns may result in an ecological fallacy [54]. Therefore, this study does not equate culture with nationality but uses the CVSCALE to measure the cultural values actually endorsed by individual respondents [17]. In this study, the Chinese and Russian samples represent different social and digital-service contexts, whereas collectivism orientation, uncertainty avoidance, and long-term orientation are treated as individual-level cultural value variables.

Beyond Hofstede’s framework, cross-cultural technology-use research has also drawn on Schwartz’s theory of cultural value orientations to explain users’ technology-related judgments and behaviors across different cultural settings through alternative value structures [55]. This line of research indicates that the cultural influences on technology use can be examined through different theoretical frameworks and further suggests that the selection of cultural dimensions should correspond to the specific research context and the mechanisms under investigation.

Of Hofstede’s six dimensions, this study selected collectivism orientation (COL), uncertainty avoidance (UAI), and long-term orientation (LTO) because they have relatively clear theoretical links to the technology acceptance relationships examined in this study. COL is associated with the group-reference and social-coordination mechanisms reflected in social influence. UAI is related to the reduction of uncertainty and the availability of external support reflected in effort expectancy and facilitating conditions. LTO is associated with the long-term functional value reflected in performance expectancy and the extended experiential value that hedonic motivation may carry in the museum context.

Power distance, masculinity/femininity, and indulgence/restraint were not included because power distance and masculinity/femininity lack direct and theoretically distinct links to the specific acceptance relationships examined in the present model. Meta-analytic evidence also indicates that their moderating effects in technology acceptance models are generally relatively weak [16]. Indulgence/restraint is not included in the original CVSCALE adopted in this study [17], and its measurement at the individual level would require a separately developed scale [56]. Moreover, this dimension reflects a general orientation toward gratification and self-restraint, whereas hedonic motivation concerns the enjoyment derived from using a specific technology. A clear and independent mechanism linking indulgence/restraint to technology-specific hedonic motivation would therefore need to be established. Based on these theoretical and measurement considerations, the present study ultimately included COL, UAI, and LTO.

### The moderating logic of collectivism (COL)

Collectivism emphasizes group relationships, shared goals, and consistency between the individual and the group. Individuals with stronger collectivist orientations are more likely to rely on social reference points and group norms when forming judgments and to regard the opinions and behavior of others as important decision cues [53]. In museum visits involving shared participation, the use of digital services is often socially visible. Visitors may judge whether a digital service is worth using based on recommendations from companions, the observable behavior of other visitors, and discussions on social media. Cross-cultural research has shown that differences in individualism–collectivism orientation can influence technology acceptance behavior [57]. A recent study of e-government AI assistants in China also identified social influence as an important predictor of usage intention [28], providing additional evidence of the relevance of social cues in Chinese digital service contexts. For visitors with stronger collectivist orientations, group norms and the behavior of others are likely to carry greater referential value. When companions, social networks, or other visitors express positive attitudes toward a digital service, these social cues are more likely to be incorporated into the individual’s acceptance judgment, thereby strengthening the positive effect of social influence on acceptance intention. Accordingly, the following hypothesis is proposed:

H2a: Collectivism (COL) positively moderates the effect of social influence (SI) on acceptance intention (BI); the higher the level of COL, the stronger the effect of SI on BI.

### The moderating logic of uncertainty avoidance (UAI)

Uncertainty avoidance reflects the extent to which individuals feel uncomfortable with ambiguity, unpredictability, and potential risk. Meta-analytic evidence indicates that UAI is related to ease-of-use judgments and technology acceptance pathways, although the direction and strength of its moderating effects may vary across technologies and model relationships [16]. In museum settings, visitors often need to understand unfamiliar interfaces and interaction procedures within a limited amount of time. Lower learning difficulty and clearer operating procedures can make the use process more predictable. For visitors with higher levels of UAI, reducing ambiguity and uncertainty during use may be particularly important. Effort expectancy may therefore become a more important basis for forming acceptance intention, strengthening its positive effect on BI.

Individuals with higher levels of UAI may also place greater importance on external support and safeguards that reduce operational risks. Stable devices and network connections, clear instructions, and on-site assistance from staff can reduce potential problems during the use of museum digital services. Previous research has shown that UAI can moderate the relationship between facilitating conditions and behavioral intention [58]. When visitors attach greater importance to the controllability and reliability of the use process, facilitating conditions are more likely to translate into acceptance intention. Therefore, the following hypotheses are proposed:

H3a: Uncertainty avoidance (UAI) positively moderates the effect of effort expectancy (EE) on acceptance intention (BI); the higher the level of UAI, the stronger the positive effect of EE on BI.
H3b: Uncertainty avoidance (UAI) positively moderates the effect of facilitating conditions (FC) on acceptance intention (BI); the higher the level of UAI, the stronger the positive effect of FC on BI.

### The moderating logic of long-term orientation (LTO)

Long-term orientation emphasizes future returns, sustained investment, and the long-term consequences of present actions [53]. Individuals with stronger long-term orientations tend to place greater value on the sustained benefits that technology may provide and may be more willing to invest the learning and adaptation effort required to obtain those benefits. Recent research has found that long-term orientation positively predicts mobile technology adoption intention [59], suggesting that when the value of a technology emerges gradually through continued use and learning, long-term value orientation may become an important basis for acceptance.

Long-term orientation may also shape how visitors evaluate the functional benefits of technology. Meta-analytic evidence indicates that long-term orientation positively moderates the relationship between perceived usefulness and behavioral intention [16]. Perceived usefulness and performance expectancy both concern users’ evaluations of the functional benefits provided by technology. In the context of museum digital services, performance expectancy involves not only whether a service improves information access and navigation during the current visit, but also whether it supports deeper cultural understanding and extends the learning experience. Visitors with stronger long-term orientations may attach greater importance to these sustained benefits, making performance expectancy more likely to translate into acceptance intention.

The moderating role of LTO in the relationship between hedonic motivation and acceptance intention is more specific to the museum context. Previous research has shown that the usability and interactivity of museum technologies can strengthen visitors’ cognitive engagement and subsequently influence their learning experiences [60]. Enjoyment generated by digital services therefore need not be limited to immediate entertainment; it may also serve as an experiential resource that supports deeper exploration, continued engagement, and cultural understanding. For visitors with stronger long-term orientations, enjoyment and immersion may be more readily translated into acceptance intention when they also contribute to further learning and sustained engagement. Accordingly, the following hypotheses are proposed:

H4a: Long-term orientation (LTO) positively moderates the effect of performance expectancy (PE) on acceptance intention (BI); the higher the level of LTO, the stronger the positive effect of PE on BI.
H4b: Long-term orientation (LTO) positively moderates the effect of hedonic motivation (HM) on acceptance intention (BI); the higher the level of LTO, the stronger the positive effect of HM on BI.

The proposed research model is shown in Fig 1.

**Fig 1:**
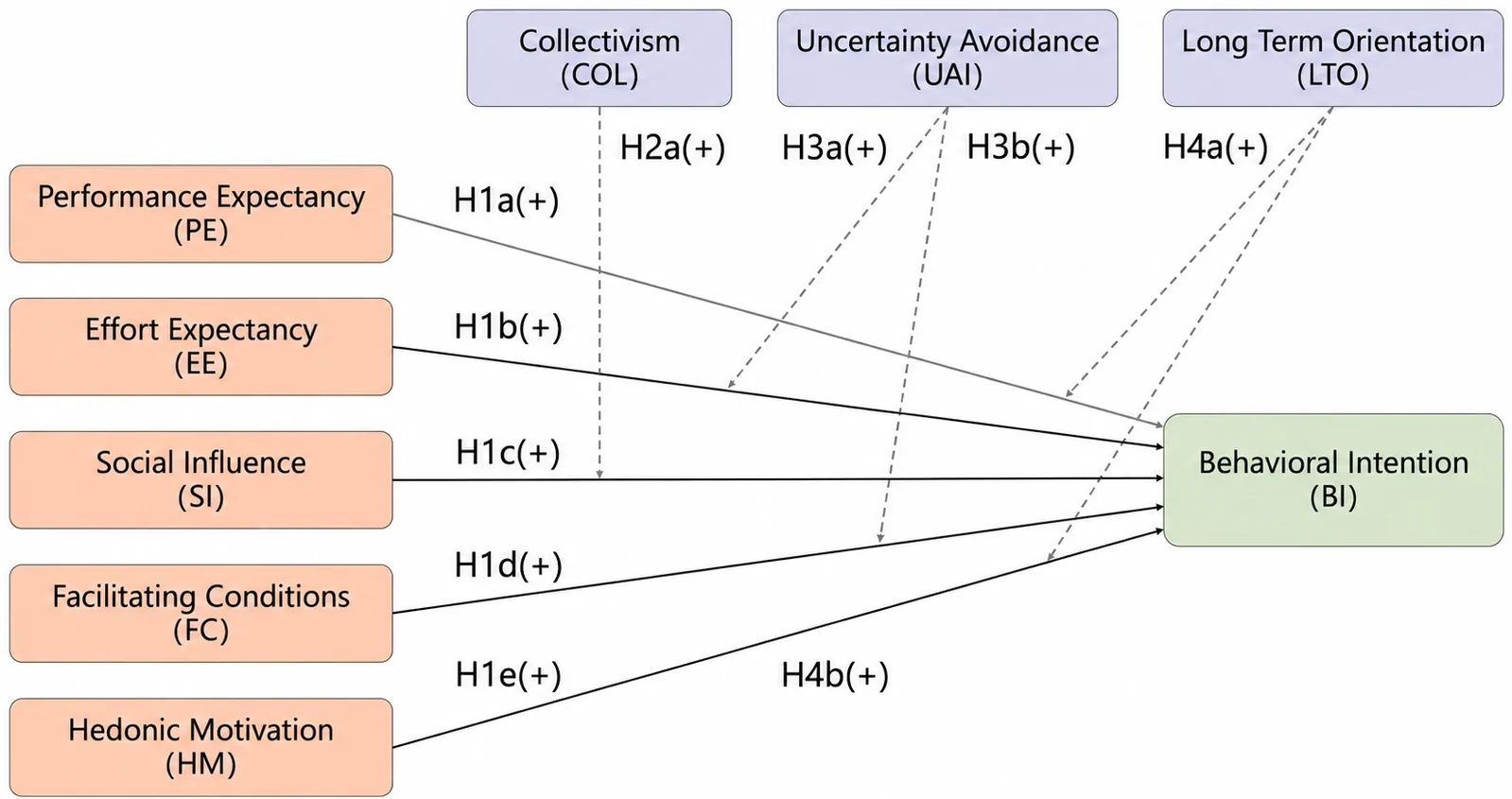
The research model.

## Materials and methods

### Questionnaire development and measurement

This study used a structured questionnaire to measure museum visitors’ acceptance intention toward digital services and its influencing factors in the Chinese and Russian samples. The items for the technology acceptance constructs (PE, EE, SI, FC, HM, and BI) were adapted from the established UTAUT2 scale [7] and were semantically adjusted to fit the context of museum digital services. For example, the technology referred to in the item wording was specified as services such as mobile guides, AR/VR experiences, and online exhibitions. The Hofstede-related cultural dimensions—collectivism orientation (COL), uncertainty avoidance (UAI), and long-term orientation (LTO)—were measured at the individual level using the CVSCALE [17] and contextualized for museum digital-service use.

To ensure consistency across language versions, the questionnaire was translated into Chinese and Russian by the first author, and the Russian version was subsequently reviewed by a Russian-language expert to ensure semantic accuracy and clarity. The item wording was revised accordingly. A small-scale pretest involving 20 participants was conducted before formal data collection to examine the clarity and comprehensibility of the questionnaire items. The questionnaire contained two parts: the first part collected respondents’ background information, and the second part included the items for each construct. All items were measured on a seven-point Likert scale ranging from 1 (strongly disagree) to 7 (strongly agree). This study was conducted in accordance with the Declaration of Helsinki and was approved by the Medical Ethics Committee of Jiangnan University (Approval No. JNU202506RB067). Before participation, all participants were informed of the study purpose, the voluntary nature of participation, the anonymous handling of data, and the use of the data solely for research purposes. Electronic informed consent was obtained from participants in the Chinese online survey, whereas oral informed consent was obtained from participants in the Russian offline survey. No personally identifiable information was collected.

### Data collection

This study used a combination of online and offline methods for data collection. The Chinese questionnaire was distributed mainly through WeChat and Xiaohongshu from 10 October 2025–20 October 2025, resulting in 350 returned questionnaires. The Russian questionnaire was collected mainly through offline intercept surveys conducted around Palace Square and university areas in Saint Petersburg from 10 October 2025–25 October 2025, resulting in 350 returned questionnaires.

To ensure data quality, this study applied strict screening criteria. First, responses were excluded if participants reported no previous experience with museum digital services, as such experience was considered necessary for evaluating acceptance intention toward these services (21 Chinese responses and 34 Russian responses). Second, invalid responses with excessively short completion times (less than 100 s) or incomplete answers were removed (13 Chinese responses and 9 Russian responses). The final valid sample included 316 Chinese questionnaires (effective rate = 90%) and 307 Russian questionnaires (effective rate = 88%). The final valid sample size in each group (N > 300) was considered sufficient for structural equation modeling (SEM) and supported stable parameter estimation.

The final sample included 316 respondents from China and 307 respondents from Russia. Men accounted for 53.48% of the Chinese sample and 58.96% of the Russian sample, while women accounted for 46.52% and 41.04%, respectively. Respondents aged 18–35 constituted the largest age group in both samples, representing 67.41% of the Chinese sample and 73.62% of the Russian sample. Participants aged 36–55 accounted for 19.94% and 18.24%, while those older than 55 accounted for 12.66% and 8.14%, respectively. Regarding education, 34.18% of the Chinese respondents had a high school education or below, 39.56% had university-level education, and 26.27% had postgraduate education or above. In the Russian sample, the corresponding proportions were 11.73%, 59.61%, and 28.66%. Respondents with university or postgraduate education accounted for 65.82% of the Chinese sample and 88.27% of the Russian sample. Detailed demographic characteristics are presented in Table 1.

**Table 1:** Participants’ demographic characteristics.

Item	Category	China (n)	China (%)	Russia (n)	Russia (%)
Gender	Male	169	53.48	181	58.96
	Female	147	46.52	126	41.04
Age	18–35	213	67.41	226	73.62
	36–55	63	19.94	56	18.24
	>55	40	12.66	25	8.14
Education	High school or below	108	34.18	36	11.73
	University	125	39.56	183	59.61
	Postgraduate or above	83	26.27	88	28.66
Total		316	100.00	307	100.00

### Data analysis

Data processing and descriptive statistical analyses were conducted using SPSS 26.0, and partial least squares structural equation modeling (PLS-SEM) was used to test the proposed model. The measurement model was assessed using indicator loadings, Cronbach’s alpha, composite reliability (CR), average variance extracted (AVE), and the heterotrait–monotrait ratio (HTMT). The structural model was evaluated using variance inflation factors (VIF), path coefficients, the coefficient of determination (R^2^), predictive relevance (Q^2^), and effect sizes (f^2^). Direct and interaction effects were assessed using 5,000 bootstrap resamples and 95% confidence intervals. Values of f^2^ equal to 0.02, 0.15, and 0.35 were interpreted as small, medium, and large effects, respectively [61].

Moderating effects were tested using a two-stage construct-score approach. Five interaction terms—SI × COL, EE × UAI, FC × UAI, PE × LTO, and HM × LTO—were created using standardized predictor and moderator scores. Significant interaction effects were further examined through simple-slope analyses at the moderator mean and at one standard deviation below and above the mean. Before comparing the Chinese and Russian samples, measurement invariance was assessed using the measurement invariance of composites (MICOM) procedure with 5,000 permutations [62]. Permutation-based multigroup analysis and PLS-MGA were subsequently used to compare the direct and interaction effects across the two samples [63,64]. Between-sample differences were evaluated using two-sided permutation p-values below 0.05 and PLS-MGA p-values below 0.05 or above 0.95.

## Results

### Measurement model

The reliability and convergent validity of the measurement model were assessed using indicator loadings, Cronbach’s alpha, composite reliability (CR), and average variance extracted (AVE). As shown in Tables 2 and 3, all indicator loadings exceeded the recommended threshold of 0.70, ranging from 0.815 to 0.923 in the Chinese sample and from 0.800 to 0.925 in the Russian sample. Cronbach’s alpha ranged from 0.828 to 0.902 in the Chinese sample and from 0.797 to 0.901 in the Russian sample, while CR values ranged from 0.890 to 0.938 and from 0.880 to 0.938, respectively. The AVE values ranged from 0.744 to 0.835 in the Chinese sample and from 0.711 to 0.834 in the Russian sample. All reliability and convergent-validity indicators met the recommended criteria, indicating satisfactory internal consistency and convergent validity in both samples.

**Table 2:** Measurement model assessment for the Chinese sample (n = 316).

Construct	Item	Indicator loading	Cronbach’s α	CR	AVE
Performance Expectancy	PE1	0.917	0.886	0.929	0.814
	PE2	0.902			
	PE3	0.887			
Effort Expectancy	EE1	0.907	0.889	0.931	0.818
	EE2	0.906			
	EE3	0.901			
Social Influence	SI1	0.885	0.873	0.921	0.795
	SI2	0.912			
	SI3	0.878			
Facilitating Conditions	FC1	0.910	0.851	0.910	0.771
	FC2	0.886			
	FC3	0.836			
Hedonic Motivation	HM1	0.912	0.894	0.934	0.825
	HM2	0.923			
	HM3	0.889			
Acceptance Intention (BI)	BI1	0.910	0.897	0.936	0.829
	BI2	0.909			
	BI3	0.912			
Collectivism Orientation	COL1	0.883	0.828	0.897	0.744
	COL2	0.858			
	COL3	0.847			
Uncertainty Avoidance	UAI1	0.900	0.864	0.917	0.786
	UAI2	0.879			
	UAI3	0.881			
Long-Term Orientation	LTO1	0.919	0.902	0.938	0.835
	LTO2	0.915			
	LTO3	0.907			

**Table 3:** Measurement model assessment for the Russian sample (n = 307).

Construct	Item	Indicator loading	Cronbach’s α	CR	AVE
Performance Expectancy	PE1	0.846	0.806	0.886	0.721
	PE2	0.838			
	PE3	0.863			
Effort Expectancy	EE1	0.921	0.872	0.921	0.797
	EE2	0.902			
	EE3	0.854			
Social Influence	SI1	0.873	0.885	0.929	0.814
	SI2	0.909			
	SI3	0.924			
Facilitating Conditions	FC1	0.800	0.823	0.894	0.738
	FC2	0.890			
	FC3	0.884			
Hedonic Motivation	HM1	0.881	0.797	0.880	0.711
	HM2	0.831			
	HM3	0.815			
Acceptance Intention (BI)	BI1	0.925	0.901	0.938	0.834
	BI2	0.912			
	BI3	0.903			
Collectivism Orientation	COL1	0.889	0.814	0.890	0.729
	COL2	0.838			
	COL3	0.834			
Uncertainty Avoidance	UAI1	0.878	0.811	0.888	0.725
	UAI2	0.818			
	UAI3	0.858			
Long-Term Orientation	LTO1	0.859	0.837	0.902	0.754
	LTO2	0.874			
	LTO3	0.872			

Discriminant validity was assessed using the heterotrait–monotrait ratio (HTMT). As shown in Table 4, all HTMT values were below the recommended threshold of 0.85. The highest HTMT value was 0.674 in the Chinese sample and 0.612 in the Russian sample, indicating satisfactory discriminant validity across all constructs in both samples.

**Table 4:** HTMT results for discriminant validity in the Chinese and Russian samples.

	PE	EE	SI	FC	HM	BI	COL	UAI	LTO
PE									
EE	0.382								
SI	0.343	0.223							
FC	0.462	0.372	0.335						
HM	0.447	0.276	0.429	0.482					
BI	0.595	0.340	0.535	0.593	0.674				
COL	0.412	0.404	0.379	0.483	0.413	0.513			
UAI	0.443	0.357	0.338	0.416	0.380	0.530	0.343		
LTO	0.313	0.186	0.273	0.347	0.293	0.345	0.168	0.245	
**Panel B. Russia (HTMT)**									
	**PE**	**EE**	**SI**	**FC**	**HM**	**BI**	**COL**	**UAI**	**LTO**
PE									
EE	0.449								
SI	0.331	0.294							
FC	0.464	0.452	0.426						
HM	0.412	0.254	0.411	0.387					
BI	0.562	0.612	0.452	0.605	0.411				
COL	0.413	0.379	0.230	0.456	0.276	0.477			
UAI	0.481	0.445	0.292	0.485	0.404	0.542	0.412		
LTO	0.453	0.354	0.330	0.459	0.455	0.517	0.365	0.433	

### Measurement invariance

Before conducting the multigroup comparisons, measurement invariance between the Chinese and Russian samples was assessed using the MICOM procedure. Configural invariance was established because the same measurement items, data treatment procedures, model specifications, and estimation settings were used in both samples. Based on 5,000 permutations, the original correlations for PE, SI, HM, COL, UAI, LTO, and BI exceeded their respective 5% quantiles, indicating compositional invariance, whereas EE and FC did not meet this criterion (Table 5, Panel A).

**Table 5:** Results of the MICOM assessment across the Chinese and Russian samples.

Panel A. Compositional invariance						
Construct	Original correlation (c)	5% quantile	Permutation (p)	Compositional invariance		
PE	0.9999	0.9987	0.733	Yes		
EE	0.9985	0.9987	0.028	No		
SI	0.9992	0.9986	0.200	Yes		
FC	0.9970	0.9985	0.004	No		
HM	0.9995	0.9981	0.427	Yes		
COL	0.9999	0.9964	0.892	Yes		
UAI	1.0000	0.9981	0.949	Yes		
LTO	0.9994	0.9980	0.430	Yes		
BI	1.0000	0.9997	0.651	Yes		
**Panel B. Equality of construct means**						
**Construct**	**Mean difference**	**2.5% quantile**	**97.5% quantile**	**Permutation (p)**	**Equal means**	
PE	0.010	−0.158	0.154	0.900	Yes	
EE	0.196	−0.164	0.158	0.014	No	
SI	0.149	−0.154	0.153	0.058	Yes	
FC	−0.109	−0.161	0.155	0.173	Yes	
HM	0.304	−0.152	0.155	<0.001	No	
COL	0.056	−0.156	0.156	0.493	Yes	
UAI	0.421	−0.159	0.154	<0.001	No	
LTO	0.327	−0.155	0.155	<0.001	No	
BI	0.146	−0.155	0.156	0.066	Yes	
**Panel C. Equality of construct variances and overall invariance**						
**Construct**	**Log variance ratio, ln(VarChina/VarRussia)**	**2.5% quantile**	**97.5% quantile**	**Permutation (p)**	**Equal variances**	**Overall invariance**
PE	0.194	−0.210	0.208	0.068	Yes	Full
EE	−0.042	−0.204	0.204	0.676	Yes	Not established
SI	−0.382	−0.283	0.271	0.009	No	Partial
FC	0.049	−0.238	0.227	0.675	Yes	Not established
HM	−0.116	−0.246	0.241	0.375	Yes	Partial
COL	−0.019	−0.221	0.225	0.866	Yes	Full
UAI	−0.271	−0.238	0.237	0.024	No	Partial
LTO	0.026	−0.251	0.246	0.842	Yes	Partial
BI	0.011	−0.242	0.247	0.931	Yes	Full

***Note.*** Results were based on 5,000 permutations. Mean differences in Panel B were calculated as China minus Russia, whereas Panel C reports the log variance ratio, ln(VarChina/VarRussia).

The subsequent tests of construct means and variances showed no significant differences for PE, COL, and BI, indicating full measurement invariance for these constructs (Table 5, Panels B and C). SI, HM, UAI, and LTO achieved compositional invariance but showed at least one significant difference in their means or variances; these constructs therefore achieved partial measurement invariance. Measurement invariance was not established for EE and FC because they failed to meet the compositional invariance criterion. Accordingly, partial or full measurement invariance provided an adequate basis for the subsequent between-sample comparisons of structural relationships involving PE, SI, HM, COL, UAI, LTO, and BI. By contrast, comparisons involving EE or FC should be regarded as exploratory and interpreted with caution because compositional invariance was not established for these constructs.

### Structural model and direct effects

Before testing the structural paths, the explanatory power, predictive relevance, and collinearity of the model were assessed. The R² values for BI were 0.777 in the Chinese sample and 0.786 in the Russian sample, with adjusted R² values of 0.767 and 0.777, respectively, indicating that the model explained a substantial proportion of the variance in acceptance intention in both samples. The corresponding Q² values were 0.597 and 0.590, both above zero, supporting the predictive relevance of the model (Table 6, Panel A). The VIF values ranged from 1.227 to 1.628 in the Chinese sample and from 1.302 to 1.930 in the Russian sample, indicating no substantial collinearity concerns (Table 6, Panel B). The structural model was therefore considered suitable for the subsequent assessment of the direct and moderating effects.

**Table 6:** Structural model assessment in the Chinese and Russian samples.

**Panel A. Explanatory power and predictive relevance**			
**Sample**	**R^2^**	**Adjusted R^2^**	**Q^2^**
China	0.777	0.767	0.597
Russia	0.786	0.777	0.590
**Panel B. Collinearity assessment**			
**Variable**	**China BI**	**Russia BI**	
PE	1.573	1.478	
EE	1.402	1.430	
SI	1.369	1.302	
FC	1.582	1.659	
HM	1.565	1.383	
COL	1.481	1.342	
UAI	1.399	1.449	
LTO	1.227	1.479	
LTO × HM	1.370	1.423	
UAI × FC	1.498	1.930	
LTO × PE	1.574	1.537	
UAI × EE	1.628	1.630	
COL × SI	1.515	1.466	

***Note.*** R^2^ = coefficient of determination; adjusted R^2^ = adjusted coefficient of determination; Q^2^ = predictive relevance; VIF = variance inflation factor.

All five direct paths were positive in both the Chinese and Russian samples, and none of the 95% confidence intervals included zero; therefore, H1a–H1e were supported in both samples (Table 7). In the Chinese sample, hedonic motivation had the largest path coefficient (β = 0.307, 95% CI [0.210, 0.382], f^2^ = 0.270), representing a medium effect. In the Russian sample, facilitating conditions (β = 0.316, 95% CI [0.231, 0.398], f^2^ = 0.282) and effort expectancy (β = 0.308, 95% CI [0.233, 0.387], f^2^ = 0.310) had the largest path coefficients, both with medium effect sizes, while the remaining direct effects were small.

**Table 7:** Direct effects in the Chinese and Russian samples.

Sample	Hypothesis	Path	β	95% CI	f^2^	p	Result
China	H1a	PE → BI	0.215	[0.128, 0.301]	0.132	<0.001	Supported
China	H1b	EE → BI	0.130	[0.052, 0.205]	0.054	0.001	Supported
China	H1c	SI → BI	0.206	[0.128, 0.280]	0.139	<0.001	Supported
China	H1d	FC → BI	0.158	[0.073, 0.245]	0.071	<0.001	Supported
China	H1e	HM → BI	0.307	[0.210, 0.382]	0.270	<0.001	Supported
Russia	H1a	PE → BI	0.213	[0.140, 0.285]	0.144	<0.001	Supported
Russia	H1b	EE → BI	0.308	[0.233, 0.387]	0.310	<0.001	Supported
Russia	H1c	SI → BI	0.202	[0.112, 0.288]	0.147	<0.001	Supported
Russia	H1d	FC → BI	0.316	[0.231, 0.398]	0.282	<0.001	Supported
Russia	H1e	HM → BI	0.133	[0.051, 0.214]	0.060	0.001	Supported

***Note.*** β represents the standardized path coefficient. Confidence intervals were obtained from 5,000 bootstrap samples. Values of f^2^ = 0.02, 0.15, and 0.35 indicate small, medium, and large effects, respectively. CI = confidence interval.

### Moderating effects and simple-slope analysis

All five interaction effects were positive in both samples, and none of their 95% confidence intervals included zero. H2a, H3a, H3b, H4a, and H4b were therefore supported in both samples (Table 8). In the Chinese sample, SI × COL, FC × UAI, and PE × LTO showed relatively larger effects, although their f² values remained small. In the Russian sample, FC × UAI showed the largest interaction effect (β = 0.221, 95% CI [0.145, 0.290], f² = 0.207), representing a medium effect, while the remaining interaction effects were small.

**Table 8:** Moderating effects of individual-level cultural values.

Sample	Hypothesis	Interaction path	β	95% CI	f²	p	Result
China	H2a	SI × COL → BI	0.144	[0.078, 0.201]	0.100	<0.001	Supported
China	H3a	EE × UAI → BI	0.089	[0.014, 0.158]	0.028	0.021	Supported
China	H3b	FC × UAI → BI	0.149	[0.060, 0.217]	0.091	<0.001	Supported
China	H4a	PE × LTO → BI	0.157	[0.063, 0.226]	0.091	0.003	Supported
China	H4b	HM × LTO → BI	0.108	[0.036, 0.188]	0.056	0.006	Supported
Russia	H2a	SI × COL → BI	0.069	[0.007, 0.131]	0.027	0.024	Supported
Russia	H3a	EE × UAI → BI	0.157	[0.086, 0.229]	0.091	<0.001	Supported
Russia	H3b	FC × UAI → BI	0.221	[0.145, 0.290]	0.207	<0.001	Supported
Russia	H4a	PE × LTO → BI	0.148	[0.069, 0.214]	0.103	<0.001	Supported
Russia	H4b	HM × LTO → BI	0.097	[0.023, 0.178]	0.040	0.009	Supported

***Note.*** β represents the standardized interaction coefficient. Confidence intervals were obtained from 5,000 bootstrap samples. Values of f^2^ = 0.02, 0.15, and 0.35 indicate small, medium, and large effects, respectively. CI = confidence interval.

Simple-slope analyses further clarified the interaction patterns. In the Chinese sample, the effects of SI, EE, FC, and PE on BI were not significant when the corresponding cultural value was low, but became significant at the mean level and increased further at the high level. The effect of HM on BI remained significant across all three levels of LTO. In the Russian sample, the effects of SI on BI and EE on BI were significant across all three moderator levels, whereas the effects of FC, PE, and HM on BI were not significant when the corresponding cultural value was low. At the mean and high levels of the moderators, all conditional effects were positive and significant (Table 9).

**Table 9:** Results of the simple-slope analyses.

Sample	Moderation relationship	Low (−1 SD), β [95% CI]	Mean, β [95% CI]	High (+1 SD), β [95% CI]
China	COL moderates SI → BI	0.056 [−0.028, 0.147]	0.203* [0.125, 0.277]	0.350* [0.240, 0.451]
China	UAI moderates EE → BI	0.043 [−0.052, 0.142]	0.133* [0.056, 0.210]	0.223* [0.113, 0.332]
China	UAI moderates FC → BI	0.012 [−0.073, 0.117]	0.160* [0.074, 0.248]	0.308* [0.167, 0.436]
China	LTO moderates PE → BI	0.066 [−0.038, 0.197]	0.220* [0.133, 0.309]	0.374* [0.234, 0.493]
China	LTO moderates HM → BI	0.198* [0.087, 0.281]	0.311* [0.213, 0.387]	0.425* [0.278, 0.546]
Russia	COL moderates SI → BI	0.134* [0.016, 0.238]	0.207* [0.115, 0.294]	0.280* [0.179, 0.385]
Russia	UAI moderates EE → BI	0.155* [0.037, 0.281]	0.312* [0.236, 0.393]	0.469* [0.383, 0.557]
Russia	UAI moderates FC → BI	0.094 [−0.008, 0.201]	0.313* [0.226, 0.396]	0.532* [0.407, 0.649]
Russia	LTO moderates PE → BI	0.064 [−0.037, 0.176]	0.212* [0.139, 0.285]	0.361* [0.259, 0.457]
Russia	LTO moderates HM → BI	0.037 [−0.054, 0.122]	0.132* [0.050, 0.213]	0.228* [0.096, 0.369]

***Note.*** Simple slopes were estimated at low (−1 SD), mean, and high (+1 SD) levels of the moderator. Confidence intervals were obtained from 5,000 bootstrap samples. An asterisk indicates that the 95% confidence interval excludes zero. CI = confidence interval; BI = acceptance intention.

The corresponding interaction patterns are shown in Figs 2 and 3.

**Fig 2:**
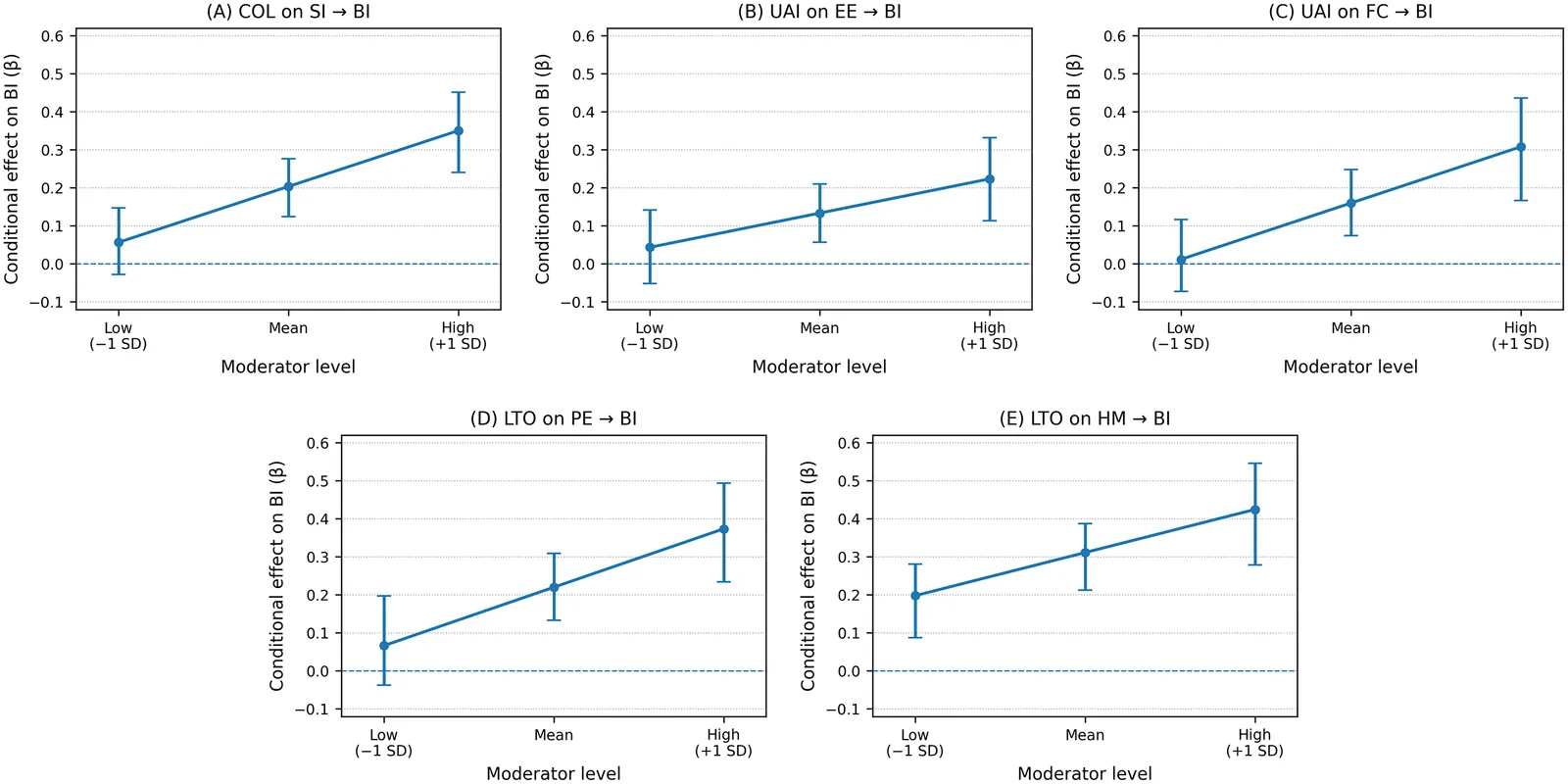
Simple-slope plots of the moderating effects in the Chinese sample.

**Fig 3:**
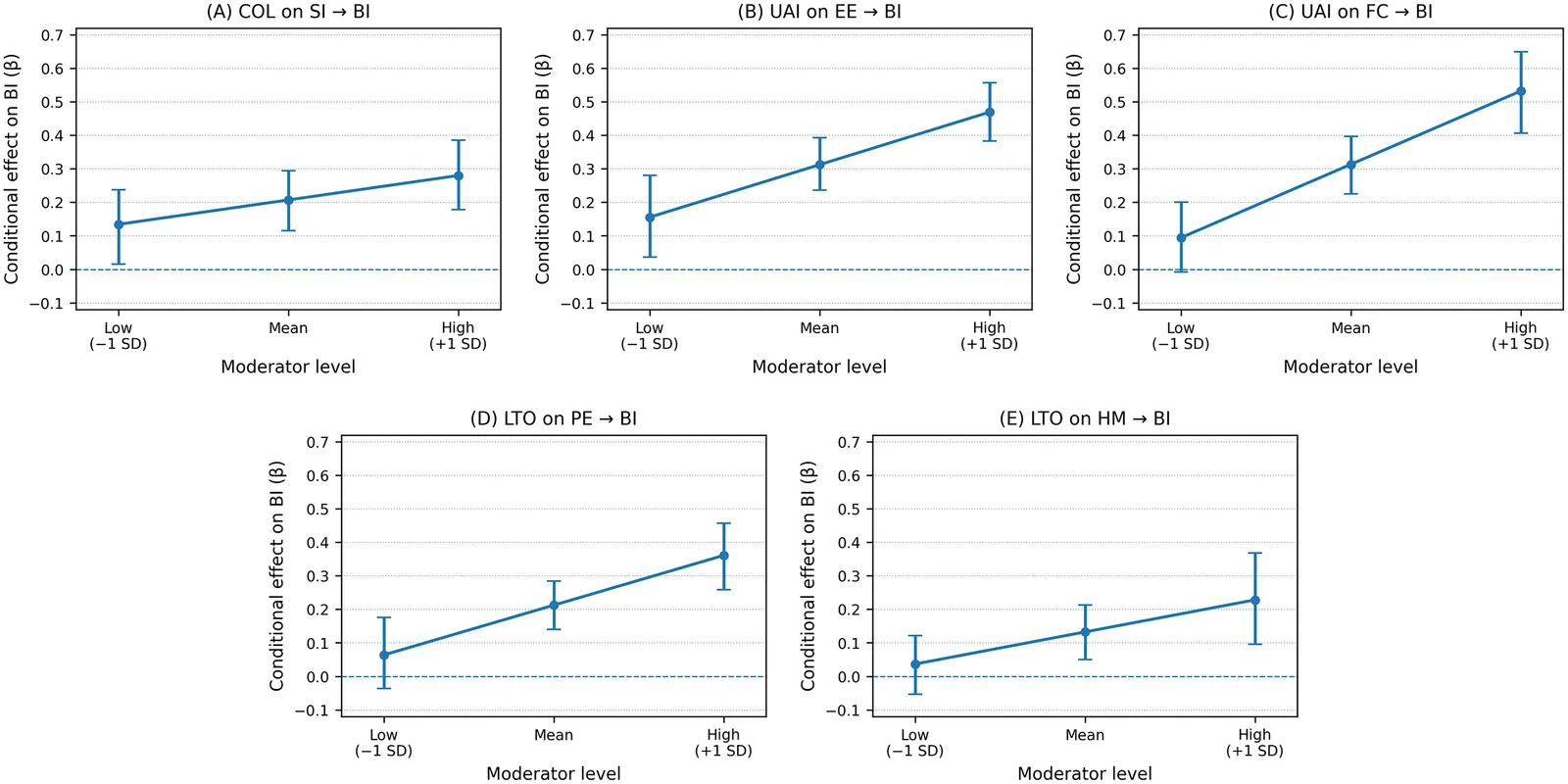
Simple-slope plots of the moderating effects in the Russian sample.

### Multigroup analysis

Permutation-based multigroup analysis and PLS-MGA yielded consistent significance decisions. The effects of EE on BI and FC on BI were stronger in the Russian sample, whereas the effect of HM on BI was stronger in the Chinese sample. No significant between-sample differences were found for PE → BI or SI → BI (Table 10). Because compositional invariance was not established for EE and FC in the MICOM assessment, the between-sample differences involving these constructs should be interpreted cautiously. In contrast, compositional invariance was established for HM, providing a firmer measurement basis for the corresponding between-sample difference. None of the five interaction paths differed significantly between the Chinese and Russian samples (Table 10). Thus, although COL, UAI, and LTO strengthened their corresponding UTAUT2 relationships within both samples, the strength of these moderating effects did not differ significantly between the two samples.

**Table 10:** Multigroup comparison of direct and interaction effects.

Effect type	Path	China β	Russia β	Difference	Permutation p	PLS-MGA p	Result
Direct	PE → BI	0.215	0.213	0.002	0.975	0.518	No significant difference
Direct	EE → BI	0.130	0.308	−0.178	0.002	0.001	Russia stronger
Direct	SI → BI	0.206	0.202	0.004	0.940	0.523	No significant difference
Direct	FC → BI	0.158	0.316	−0.157	0.018	0.005	Russia stronger
Direct	HM → BI	0.307	0.133	0.174	0.013	0.996	China stronger
Interaction	SI × COL → BI	0.144	0.069	0.075	0.167	0.940	No significant difference
Interaction	EE × UAI → BI	0.089	0.157	−0.068	0.233	0.090	No significant difference
Interaction	FC × UAI → BI	0.149	0.221	−0.073	0.206	0.074	No significant difference
Interaction	PE × LTO → BI	0.157	0.148	0.008	0.909	0.534	No significant difference
Interaction	HM × LTO → BI	0.108	0.097	0.010	0.885	0.578	No significant difference

***Note.*** Differences were calculated as China minus Russia. For the permutation test, p-values below 0.05 indicate a significant between-sample difference. For PLS-MGA, p-values below 0.05 or above 0.95 indicate a significant between-sample difference. PE = performance expectancy; EE = effort expectancy; SI = social influence; FC = facilitating conditions; HM = hedonic motivation; COL = collectivism orientation; UAI = uncertainty avoidance; LTO = long-term orientation; BI = acceptance intention.

## Discussion

To address RQ1 and RQ2, this study examined the effects of the core UTAUT2 variables on museum visitors’ acceptance intention toward digital services (BI) in the Chinese and Russian samples. Performance expectancy, effort expectancy, social influence, facilitating conditions, and hedonic motivation all had significant positive effects in both samples, although the strength of several relationships differed between them. The relatively high R² values may partly reflect the model’s broad specification, which includes five direct predictors and five interaction terms; they indicate strong explanatory power within the present samples but should not be interpreted as a complete causal explanation or assumed to generalize unchanged to other contexts.

Performance expectancy consistently promoted acceptance intention in both samples, with nearly identical path coefficients (China: β = 0.215; Russia: β = 0.213). The PLS-MGA results likewise showed no significant difference between the two groups. This finding is consistent with the central premise of UTAUT2: users are more likely to accept a technology when they perceive clear practical benefits from its use [7]. In the museum context, these benefits include helping visitors understand exhibition content, obtain relevant background information, and improve the efficiency of their visits. Previous digital museum research has similarly identified functional and informational value as important determinants of user acceptance and continuance intention [13,65]. The result also aligns with findings from other technology adoption contexts, where performance expectancy has generally shown a stable predictive effect [8,46].

Effort expectancy and facilitating conditions significantly influenced acceptance intention in both samples, but their effects were stronger in the Russian sample (EE → BI: China β = 0.130, Russia β = 0.308; FC → BI: China β = 0.158, Russia β = 0.316). The PLS-MGA results showed significant between-group differences for both paths. However, EE and FC did not establish compositional invariance in Step 2 of MICOM. These differences should therefore be treated as exploratory findings and interpreted with caution rather than being directly attributed to national cultural differences [62]. During on-site visits, visitors often need to decide within a limited period whether a digital service is easy to operate, whether the system is stable, and whether adequate assistance is available. Ease of use and external support may therefore directly affect their willingness to try such services [66]. Recent research has also shown that perceived ease of use improves museum visitors’ perceptions of the usefulness of AR and their attitudes toward adopting it [67]. By contrast, Liang and Wang [48] found that EE and FC did not significantly affect continuance intention in a lightweight digital museum platform. The importance of these factors may therefore vary with service complexity, the use setting, and the forms of support available.

Social influence significantly promoted acceptance intention in both samples, with almost identical path coefficients (China: β = 0.206; Russia: β = 0.202) and no significant between-group difference. In on-site museum settings, peer recommendations, information shared on social media, guidance provided by the museum, and the visible use of digital services by other visitors may all serve as reference cues when visitors decide whether to adopt such services. This result is consistent with findings from several studies of virtual reality and museum digital displays [68,69]. Other studies, however, have reported no significant effect of social influence on intention to use digital museums [13,26]. This inconsistency may be related to the use context. During on-site visits, where time and attention are limited, visitors may rely more heavily on the behavior and evaluations of others to reduce decision costs. In online environments, where users are more familiar with the technology and have greater autonomy, social influence may play a weaker role.

Hedonic motivation significantly promoted acceptance intention in both samples, but its effect was substantially stronger in the Chinese sample (China: β = 0.307; Russia: β = 0.133), and the between-group difference was significant. Because HM established compositional invariance, this result has a stronger basis for group comparison. It indicates that, in the Chinese sample, enjoyment generated by interactivity and immersive experiences was more readily translated into acceptance intention toward museum digital services. Previous studies have identified enjoyment, immersion, and entertainment as important drivers of digital cultural heritage experiences [70–72]. Shi et al. [73] further found that perceived enjoyment not only directly promoted continuance intention toward a digital museum but also mediated the effects of interactivity and vividness on continuance intention. However, Tan et al. [74] reported that hedonic motivation influenced experience satisfaction but did not directly affect continuance intention. These findings suggest that the role of hedonic motivation may vary according to the use context and the outcome variable examined. Although the present study confirmed that the HM → BI relationship was significantly stronger in the Chinese sample, it did not directly test the mechanism underlying this difference. The result should therefore be interpreted as a difference in path strength rather than attributed to a specific cultural characteristic.

To address RQ3, this study examined the moderating effects of collectivism orientation (COL), uncertainty avoidance (UAI), and long-term orientation (LTO). The three individual cultural values moderated five relationships, but none of the moderating effects differed significantly between the two samples. Individual cultural values therefore shaped the extent to which visitors relied on different decision cues, while the overall pattern of these effects remained similar across the Chinese and Russian samples.

Collectivism orientation strengthened the effect of social influence on acceptance intention in both samples. Although the moderating coefficient was higher in the Chinese sample (China: β = 0.144; Russia: β = 0.069), the between-group difference was not significant. For visitors with stronger collectivist orientations, peer opinions, museum guidance, and the visible atmosphere of on-site use were more likely to inform acceptance judgments. This finding is broadly consistent with previous studies that have incorporated cultural factors into technology acceptance frameworks. Individuals with stronger collectivist orientations are more likely to rely on social reference points when evaluating technology adoption, thereby strengthening the effect of social influence [30]. Meta-analytic evidence also indicates that cultural values can alter the strength of key relationships in technology acceptance models and that social cues do not operate in the same way across cultural contexts [75].

Uncertainty avoidance strengthened the effects of effort expectancy and facilitating conditions on acceptance intention in both samples. Visitors who are more sensitive to uncertainty therefore place greater emphasis on whether operating procedures are clear and whether adequate technical and on-site support is available [58]. Museum digital services are often used through relatively unfamiliar interfaces, within limited visit times, and in complex on-site environments. When visitors cannot easily anticipate the outcome of an operation or obtain timely assistance with devices, connectivity, or staff support, their willingness to use the service may be more easily affected. Although both moderating coefficients were higher in the Russian sample, the PLS-MGA results showed no significant between-group differences. Moreover, because EE and FC did not establish compositional invariance, the relative strength of these moderating effects should not be further compared across the two samples [62].

Long-term orientation strengthened the effects of both performance expectancy and hedonic motivation on acceptance intention in the two samples. The positive moderation of the PE → BI relationship indicates that, for visitors who place greater emphasis on long-term benefits, the capacity of digital services to support continued learning, deepen cultural understanding, and extend the value of a museum visit becomes more important in acceptance decisions. The positive moderating effect of long-term orientation on the HM → BI relationship also indicates that visitors’ evaluations of digital experiences are not limited to immediate enjoyment. For visitors with stronger long-term orientations, interactivity, immersion, and enjoyment may provide more than entertainment; they may also be seen as effective ways to sustain engagement, enrich the museum experience, and deepen cultural understanding. Hedonic value and long-term value are therefore not necessarily opposed in the museum context. When enjoyable experiences support continued participation and cultural learning, their value is more likely to be recognized by visitors with stronger long-term orientations. Meta-analytic evidence likewise suggests that cultural values such as long-term orientation can alter the strength of key relationships in technology acceptance models [16]. However, the PLS-MGA results showed no significant between-group differences in either the LTO × PE → BI or LTO × HM → BI relationship. These moderating effects are therefore better understood as individual-level cultural mechanisms shared by the Chinese and Russian samples rather than as patterns specific to either national sample.

## Conclusions

This study integrated a context-adapted UTAUT2 model with three individual cultural values—collectivism orientation, uncertainty avoidance, and long-term orientation—to examine museum visitors’ acceptance intention toward digital services in China and Russia. Performance expectancy, effort expectancy, social influence, facilitating conditions, and hedonic motivation all had significant positive effects in both samples, supporting the explanatory value of UTAUT2 in the museum digital service context.

The multigroup analysis further showed no significant between-group differences in the effects of performance expectancy or social influence. Hedonic motivation had a stronger effect in the Chinese sample, whereas effort expectancy and facilitating conditions had stronger effects in the Russian sample, although the latter two differences should be interpreted in light of the measurement invariance results. Collectivism orientation, uncertainty avoidance, and long-term orientation moderated five relationships, but none of these moderating effects differed significantly between the two samples.

Overall, acceptance intention toward museum digital services is jointly shaped by functional benefits, ease of use, social cues, facilitating conditions, and experiential value, as well as by visitors’ individual cultural value orientations. The findings identify several sample-specific differences in the direct relationships while also revealing acceptance mechanisms shared across the two samples.

### Contributions and implications

Theoretically, this study applies a context-adapted UTAUT2 model to museum digital services and examines mobile applications, online exhibitions, and AR/VR-based guides as forms of overall digital support. Rather than using nationality as a direct proxy for culture, it measures collectivism orientation, uncertainty avoidance, and long-term orientation at the individual level. This provides a more specific account of how cultural values shape visitors’ reliance on social cues, operating conditions, and experiential value. The study also combines MICOM with PLS-MGA to formally compare the two samples and distinguish numerical differences in path coefficients from statistically supported between-group differences. This approach strengthens the rigor of the cross-sample analysis and provides clearer evidence of both shared mechanisms and sample-specific patterns in museum digital service acceptance.

In practical terms, museums can optimize digital services according to visitors’ primary concerns. Greater interactivity, immersion, and narrative engagement may benefit visitors who place more value on experience. Clearer operating procedures and reliable devices, connectivity, and on-site assistance may be more important for visitors who are concerned with ease of use and support. Museums may also increase the social visibility of digital services through demonstrations, shared participation, and shareable experiences, while making their value for continued learning, cultural understanding, and extended engagement more explicit.

### Limitations and future research

This study leaves several opportunities for further research. First, the samples were drawn from China and Russia, and different data collection approaches were used in the two countries. This may have affected sample comparability to some extent. Future studies could adopt more closely aligned survey procedures and include additional countries, regions, and museum types.

Second, EE and FC did not establish compositional invariance in Step 2 of MICOM. Between-group comparisons involving these constructs should therefore be interpreted with caution. Future research could refine the relevant measurement items and repeat the comparison after stronger measurement invariance has been established.

In addition, the study primarily relied on cross-sectional survey data and acceptance intention and examined different forms of museum digital services within an overall service context. Future research could incorporate actual usage data, longitudinal surveys, or on-site observation and examine specific digital service types separately.

## Supporting information

S1 Data De-identified item-level survey dataset for the Chinese and Russian samples, with a variable codebook. (XLSX)

S1 File Background variables and questionnaire items used in the analysis, including the Chinese and Russian versions and an English reference translation. (DOCX)
