# Supplementary material for: Museum visitors’ acceptance of digital services in China and Russia: Extending UTAUT2 with individual-level cultural values
Source: PLoS One. 2026 Nov 2;21(11):e0357563. doi: 10.1371/journal.pone.0357563 (PMC13726987; doi:10.1371/journal.pone.0357563)
Supplement: Supplementary file 1 [file pone.0357563.s002.docx]

S1 File. Questionnaire items used in the analysis

Note. This file contains the background variables and measurement items included in the analytic dataset, together with an English reference translation. The survey was administered in Chinese in China and in Russian in Russia. The English translation was not administered to participants.

# English reference translation

English reference translation. The survey was administered in Chinese in China and in Russian in Russia. The English text below is provided for transparency and was not administered to participants. Only the background variables and measurement items included in the analytic dataset are presented.

Dear visitor, I am a student specializing in museum studies and am conducting a survey on factors influencing interactive experiences with digital services. Thank you for answering the following questions. Your information will be kept strictly confidential, and your responses will be used only for research purposes and not for commercial purposes. There are no right or wrong answers. Please answer according to your actual experience. Thank you for your support.

## Part 1. Background questions

| **No.** | **Question** | **Response options** |
| --- | --- | --- |
| 1 | Gender | Male; Female |
| 2 | Age | 18–35; 36–55; >55 |
| 3 | Highest level of education completed or currently pursued | High school or below; University; Postgraduate or above |
| 4 | Occupation | Student; Company employee; Civil servant/public institution employee; Self-employed; Other |
| 5 | Main way of visiting museums | Alone; With family/friends; Tour group; School/institution-organized visit |
| 6 | Have you used museum digital services? | Yes; No |

## Part 2. Measurement items

Instruction: Please indicate your agreement with each statement using a seven-point Likert scale ranging from 1 (strongly disagree) to 7 (strongly agree). Museum digital services include mobile guides, AR/VR experiences, interactive screens, digital collections, online exhibitions, and exhibition applications.

| **Item** | **Statement** |
| --- | --- |
| **PE1** | Using digital services (e.g., AR guides) significantly deepens my understanding of the historical background of exhibits. |
| **PE2** | Digital services help me notice exhibition details that are easily overlooked during a traditional visit. |
| **PE3** | Digital services increase my overall interest in museum exhibition content. |
| **EE1** | The interface design of digital services allows me to find the functions I need easily (e.g., QR-code interpretation or map navigation). |
| **EE2** | Learning to use museum digital tools does not require too much effort. |
| **EE3** | The operating procedures of digital services are intuitive to me. |
| **SI1** | Recommendations from companions or friends interested in culture significantly influence my use of digital services. |
| **SI2** | Demonstrations of digital services by museum staff increase my willingness to use them. |
| **SI3** | When I see other visitors using digital services, I am more willing to try them. |
| **FC1** | I have sufficient technical resources (e.g., a mobile phone, internet access, or venue equipment) to use museum digital services. |
| **FC2** | Self-service text, image, or video instructions can effectively solve problems I encounter when using digital services. |
| **FC3** | On-site staff assistance makes me feel more confident when using digital services. |
| **HM1** | The entertaining design of digital services (e.g., artifact reconstruction animations or puzzle games) makes the visit more enjoyable. |
| **HM2** | Using digital services is an enjoyable part of the museum visit. |
| **HM3** | The novelty of digital interaction makes me feel excited. |
| **BI1** | I intend to continue using digital services when visiting museums in the future. |
| **BI2** | If sufficient equipment support is available, I will actively choose digital services rather than traditional guides. |
| **BI3** | I will recommend the museum’s digital services to my friends. |
| **COL1** | During museum visits, I place greater value on digital services that promote interaction with family or friends (e.g., shared screens or multi-user interactive projections). |
| **COL2** | I prefer digital services that support group participation (e.g., collaborative multi-user AR puzzles). |
| **COL3** | To improve my companions’ experience, I am willing to adjust how I use digital devices. |
| **UAI1** | I tend to follow the operating procedures of digital services strictly (e.g., scanning QR codes step by step). |
| **UAI2** | Before using a newly introduced museum digital function, I need to confirm its information accuracy and stability. |
| **UAI3** | Clear operating instructions are essential for my successful use of digital services. |
| **LTO1** | I believe museum digital education resources are worth repeated study. |
| **LTO2** | I am willing to spend time learning complex digital tools (e.g., 3D artifact-modeling software). |
| **LTO3** | I believe that mastering museum-related digital skills is beneficial for future cultural learning. |

# Chinese questionnaire（中文版问卷）

亲爱的游客：您好！我是一名博物馆专业的学生，我正在对数字服务交互体验影响因素进行调查，感谢您就下列问题作答，您的信息都将受到严格保密，您的回答仅作研究之用，不涉及商业用途，所有回答无对错之分，请您根据实际情况放心填写。感谢您的帮助与支持！

## 第一部分：背景信息

| **No.** | **Question** | **Response options** |
| --- | --- | --- |
| 1 | 您的性别是 | 男；女 |
| 2 | 年龄阶段 | 18–35；36–55；>55 |
| 3 | 您正在攻读或已经获得的最高学历 | 高中及以下；本科；硕士及以上 |
| 4 | 您的职业 | 学生；企业员工；公务员/事业单位；个体经营者；其他 |
| 5 | 参观博物馆的主要方式 | 独自参观；与家人/朋友同行；旅行团；学校/机构组织 |
| 6 | 是否使用过博物馆数字服务 | 是；否 |

## 第二部分：测量题项

说明：本问卷旨在了解您对博物馆数字服务（如导览APP、VR/AR体验、在线展览等）的态度。答案无对错之分，请按真实感受填写。所有题项均采用七点李克特量表，1表示“非常不同意”，7表示“非常同意”。

| **Item** | **Statement** |
| --- | --- |
| **PE1** | 使用数字服务（如AR导览）显著加深了我对展品历史背景的理解 |
| **PE2** | 数字服务帮助我发现了传统参观中容易忽略的展览细节 |
| **PE3** | 数字服务提升了我对博物馆展览内容的整体兴趣 |
| **EE1** | 数字服务的界面设计让我能轻松找到所需功能（如扫码讲解、地图导航） |
| **EE2** | 学习使用博物馆的数字工具不需要花费我太多精力 |
| **EE3** | 数字服务的操作流程符合我的直觉习惯 |
| **SI1** | 同行参观者或文化圈内朋友的推荐会显著影响我使用数字服务 |
| **SI2** | 博物馆工作人员对数字服务的演示会增加我的使用意愿 |
| **SI3** | 看到其他观众使用数字服务时，我会更愿意尝试 |
| **FC1** | 我有足够的技术支持（手机/网络/场馆设备）使用博物馆数字服务 |
| **FC2** | 图文/视频自助指南能有效解决我使用数字服务时遇到的问题 |
| **FC3** | 现场工作人员的协助让我使用数字服务时更安心 |
| **HM1** | 数字服务的趣味性设计（如文物复原动画/解谜游戏）让参观更享受 |
| **HM2** | 使用数字服务是整个参观过程中令人愉悦的环节 |
| **HM3** | 数字交互的新颖性让我感到兴奋 |
| **BI1** | 未来参观博物馆时，我会继续使用数字服务 |
| **BI2** | 若有足够设备支持，我会主动选择数字服务而非传统导览 |
| **BI3** | 我会向朋友推荐该博物馆的数字服务 |
| **COL1** | 在博物馆参观中，我更看重能促进家人或朋友间互动交流的数字服务（如共享屏幕、多人互动投影） |
| **COL2** | 我会优先选择支持多人共同参与的数字服务（如多人AR合作解谜） |
| **COL3** | 为使同行同伴获得更好体验，我愿意调整自己使用数字设备的方式 |
| **UAI1** | 我倾向于严格遵守数字服务的操作流程（如按步骤扫码） |
| **UAI2** | 使用博物馆新推出的数字功能前，我需要确认其信息准确性和稳定性 |
| **UAI3** | 清晰的操作指引对我能否顺利使用数字服务至关重要 |
| **LTO1** | 我认为博物馆的数字教育资源值得反复学习研究 |
| **LTO2** | 我愿意花费时间学习复杂的数字工具（如3D文物建模软件） |
| **LTO3** | 我认为掌握博物馆数字技能对未来文化学习有益 |

# Russian questionnaire (Русская версия анкеты)

Уважаемый посетитель! Я студент музейного профиля, изучающий факторы взаимодействия с цифровыми услугами. Ваши ответы конфиденциальны и используются исключительно в научных целях. Ответы не имеют правильных вариантов — пожалуйста, отвечайте искренне. Благодарим за участие!

## Часть 1. Демографические и фоновые вопросы

| **No.** | **Question** | **Response options** |
| --- | --- | --- |
| 1 | Пол | Мужской; Женский |
| 2 | Возраст | 18–35; 36–55; >55 |
| 3 | Образование | Среднее/ниже; Бакалавр; Магистр/выше |
| 4 | Профессия | Студент; Работник компании; Госслужащий; Индивидуальный предприниматель; Другое |
| 5 | Формат посещения музея | Один; С семьёй/друзьями; С тургруппой; С учебным/рабочим коллективом |
| 6 | Использовали цифровые услуги музеев? | Да; Нет |

## Часть 2. Оценка цифровых услуг

Инструкция: Оцените степень согласия с каждым утверждением по семибалльной шкале, где 1 = «Совершенно не согласен», 4 = «Нейтрально», 7 = «Полностью согласен». Цифровые музейные сервисы включают мобильные гиды, AR/VR-аттракционы, интерактивные экраны, цифровые коллекции, онлайн-выставки и приложения для экскурсий.

| **Item** | **Statement** |
| --- | --- |
| **PE1** | Использование цифровых услуг (напр., AR-гида) углубило моё понимание исторического контекста экспонатов |
| **PE2** | Цифровые услуги помогли заметить детали, упускаемые при традиционном осмотре |
| **PE3** | Цифровые услуги повысили мой общий интерес к экспозиции |
| **EE1** | Интерфейс позволяет легко находить функции (навигация, сканирование QR) |
| **EE2** | Обучение использованию инструментов не требует больших усилий |
| **EE3** | Операционные процессы интуитивно понятны |
| **SI1** | Рекомендации других посетителей или знатоков культуры влияют на мой выбор |
| **SI2** | Демонстрация услуг сотрудниками музея повышает мою готовность к использованию |
| **SI3** | Наблюдение за другими посетителями стимулирует моё желание попробовать |
| **FC1** | У меня есть необходимые ресурсы (телефон/интернет/оборудование) |
| **FC2** | Графические/видеоинструкции эффективно решают мои проблемы |
| **FC3** | Помощь сотрудников на месте даёт мне уверенность |
| **HM1** | Игровые элементы (анимация, квесты) делают посещение увлекательнее |
| **HM2** | Использование услуг — один из самых приятных этапов посещения |
| **HM3** | Новизна цифрового взаимодействия вызывает у меня интерес |
| **BI1** | В будущем я продолжу использовать цифровые услуги |
| **BI2** | При возможности я выберу их вместо традиционных аудиогидов |
| **BI3** | Я порекомендую эти услуги друзьям |
| **COL1** | В музее я больше ценю цифровые сервисы, которые позволяют взаимодействовать с семьёй или друзьями (например, совместный просмотр на экране или интерактивные проекции для группы). |
| **COL2** | Я выбираю услуги с возможностью группового участия (напр., совместные AR-головоломки) |
| **COL3** | Ради лучшего опыта спутников я готов адаптировать своё использование устройств |
| **UAI1** | Я строго следую инструкциям (напр., пошаговое сканирование QR-кодов) |
| **UAI2** | Перед использованием новых функций я проверяю их точность и стабильность |
| **UAI3** | Чёткие руководства критически важны для меня |
| **LTO1** | Цифровые образовательные ресурсы музея заслуживают глубокого изучения |
| **LTO2** | Я готов учиться сложным инструментам (напр., ПО для 3D-моделирования) |
| **LTO3** | Владение цифровыми навыками полезно для будущего культурного развития |

# Measurement sources and coding notes

| **Constructs** | **Item codes** | **Source and adaptation** |
| --- | --- | --- |
| Performance expectancy; effort expectancy; social influence; facilitating conditions; hedonic motivation; behavioral intention | PE1–PE3; EE1–EE3; SI1–SI3; FC1–FC3; HM1–HM3; BI1–BI3 | Adapted and contextualized for museum digital services from Venkatesh V, Thong JYL, Xu X. Consumer acceptance and use of information technology: Extending the unified theory of acceptance and use of technology. MIS Quarterly. 2012;36(1):157–178. |
| Collectivism orientation; uncertainty avoidance; long-term orientation | COL1–COL3; UAI1–UAI3; LTO1–LTO3 | Adapted and contextualized for museum digital services from Yoo B, Donthu N, Lenartowicz T. Measuring Hofstede’s five dimensions of cultural values at the individual level: Development and validation of CVSCALE. Journal of International Consumer Marketing. 2011;23(3–4):193–210. doi:10.1080/08961530.2011.578059. |

**Coding note.** All measurement items were scored from 1 to 7. Higher scores indicate higher levels of the corresponding construct. No reverse-scored items were used. The labels COL1–COL3 are used consistently in the manuscript and supporting files.
